# The role of complement factor I rare genetic variants in age related macular degeneration in Finland

**DOI:** 10.1093/hmg/ddae165

**Published:** 2024-11-25

**Authors:** Anneliza Andreadi, Thomas M Hallam, Vicky Brocklebank, Scott J Sharp, Patrick R Walsh, Tom Southerington, Marco Hautalahti, David H Steel, Andrew J Lotery, Claire L Harris, Kevin J Marchbank, David Kavanagh, Amy V Jones

**Affiliations:** Complement Therapeutics Research Group, Translational and Clinical Research Institute, Newcastle University, Framlington Place Newcastle upon Tyne, NE2 4HH, United Kingdom; National Renal Complement Therapeutics Centre, Royal Victoria Infirmary, Queen Victoria Road, Newcastle upon Tyne, NE1 4LP, United Kingdom; Gyroscope Therapeutics Limited, A Novartis Company, Rolling Stock Yard, 188 York Way, London, N7 9AS, United Kingdom; Complement Therapeutics Research Group, Translational and Clinical Research Institute, Newcastle University, Framlington Place Newcastle upon Tyne, NE2 4HH, United Kingdom; National Renal Complement Therapeutics Centre, Royal Victoria Infirmary, Queen Victoria Road, Newcastle upon Tyne, NE1 4LP, United Kingdom; Gyroscope Therapeutics Limited, A Novartis Company, Rolling Stock Yard, 188 York Way, London, N7 9AS, United Kingdom; Complement Therapeutics Research Group, Translational and Clinical Research Institute, Newcastle University, Framlington Place Newcastle upon Tyne, NE2 4HH, United Kingdom; National Renal Complement Therapeutics Centre, Royal Victoria Infirmary, Queen Victoria Road, Newcastle upon Tyne, NE1 4LP, United Kingdom; Finnish Biobank Cooperative Regus, Yliopistonkatu 31, 20100 Turku, Finland; University of Turku, Hospital District of Southwest Finland, Finnish Biobank Cooperative – FINBB, Turku, Finland; Finnish Biobank Cooperative Regus, Yliopistonkatu 31, 20100 Turku, Finland; Biosciences Institute, Newcastle University, International Centre for Life, Central Parkway, Newcastle upon Tyne, NE1 3BZ, United Kingdom; Sunderland Eye Infirmary, Queen Alexandra Rd, Sunderland, SR2 9HP, United Kingdom; Clinical and Experimental Sciences, Faculty of Medicine, Southampton General Hospital, University of Southampton, Southampton, SO17 1BJ, United Kingdom; Complement Therapeutics Research Group, Translational and Clinical Research Institute, Newcastle University, Framlington Place Newcastle upon Tyne, NE2 4HH, United Kingdom; Gyroscope Therapeutics Limited, A Novartis Company, Rolling Stock Yard, 188 York Way, London, N7 9AS, United Kingdom; National Renal Complement Therapeutics Centre, Royal Victoria Infirmary, Queen Victoria Road, Newcastle upon Tyne, NE1 4LP, United Kingdom; Gyroscope Therapeutics Limited, A Novartis Company, Rolling Stock Yard, 188 York Way, London, N7 9AS, United Kingdom; Complement Therapeutics Research Group, Translational and Clinical Research Institute, Newcastle University, Framlington Place Newcastle upon Tyne, NE2 4HH, United Kingdom; National Renal Complement Therapeutics Centre, Royal Victoria Infirmary, Queen Victoria Road, Newcastle upon Tyne, NE1 4LP, United Kingdom; Gyroscope Therapeutics Limited, A Novartis Company, Rolling Stock Yard, 188 York Way, London, N7 9AS, United Kingdom

**Keywords:** age related macular degeneration, complement, factor I, aHUS, FINBB, Finland, rare genetic variants

## Abstract

Age-related macular degeneration (AMD) is the leading cause of irreversible blindness in the developed world. The alternative pathway (AP) of complement has been linked to the pathogenesis of AMD. In particular, rare variants (RVs) in the complement factor I (*CFI*) gene encoding the Factor I (FI) protein confer increased AMD risk. The prevalence of *CFI* RVs are well characterised in European AMD, however little is known about other populations. The Finnish population underwent genetic restriction events which have skewed allele frequencies in unexpected ways. A series of novel or enriched *CFI* RVs were identified in individuals with dry AMD from the Finnish Biobank Cooperative (FINBB), but the relationship between these genotypes and contribution to disease was unclear. Understanding how RVs impact the ability of FI to regulate the complement system is important to inform mechanistic understanding for how different genotypes contribute to disease development. To explore this a series of *in vitro* assays were used to functionally characterise the protein products of 3 *CFI* RVs enriched in FINBB dry AMD, where no prior data were available. The G547R variant resulted in almost complete loss of both classical pathway and AP regulatory potential. The c.982 g>a variant encoding G328R FI perturbed an exon splice enhancer site which resulted in exon skipping and a premature stop codon *in vitro* and low levels of FI *in vivo.* Despite detailed analysis no defect in levels or function was demonstrated in T107A. Functional characterization of all Finnish *CFI* RVs in the cohort allowed us to demonstrate that in Finnish dry AMD, collectively the type 1 *CFI* RVs (associated with FI haploinsufficiency) were significantly enriched with odds ratio (ORs) of 72.6 (95% confidence interval; CI 16.92 to 382.1). Meanwhile, type 2 *CFI* RVs (associated with FI dysfunction) collectively conferred a significant OR of 4.97 (95% CI 1.522 to 15.74), and non-impaired or normal *CFI* RV collectively conferred an of OR 3.19 (95% CI 2.410 to 4.191) although this was driven primarily by G261D. Overall, this study for the first time determined the ORs and functional effect for all *CFI* RVs within a Geographic Atrophy (GA) cohort, enabling calculations of combined risk scores that underline the risk conferred by type 1 and 2 CFI RVs in GA/AMD.

## Introduction

Age-related macular degeneration (AMD) is the commonest cause of irreversible vision loss in the developed world [1]. Genome-wide association studies first identified the role of the complement system in the pathogenesis of disease in 2005 [2–4]. Single nucleotide polymorphism association studies identified common genetic variants in complement factor I (*CFI*) that increased risk for AMD [5] with next-generation sequencing studies subsequently demonstrating an increased burden of rare *CFI* variants in disease [6–8].

Initial interrogation demonstrated that rare variants (RVs) in *CFI* associated with low factor I (FI) levels (type 1 variants) were a strong driver of AMD risk [9–13]. Carriers of type 1 *CFI* RVs have an earlier age of onset and disease progression [14–18] and increased incidence of reticular pseudodrusen [12]. Presence of type 1 *CFI* RVs have been linked to a thinner retinal pigment epithelium cell layer and Bruch’s membrane complex in both healthy individuals and those with AMD when analysed using retinal optical coherence tomography imaging, although this difference only emerges later in life, likely reflecting an accelerated ageing process [10].

Recent approvals for two complement inhibitory therapeutics (pegcetacoplan Syfovre, Apellis; avacincaptad pegol, Izervay, Astellas) herald a new era for the treatment of late geographic atrophy (GA) in AMD, however their anatomical and functional benefit appear to be limited [19]. Although the complement system is strongly implicated in the pathogenesis of AMD, genetic studies have also identified other pathways which play a major contributory role. The age-related maculopathy susceptibility 2/high-temperature requirement A serine peptidase 1 (*ARMS2-HTRA1*) locus is also a strong risk factor for AMD. Here, the functional mechanism is thought to be mediated through overexpression of *HTRA1* leading to accumulation of macrophages in the subretinal space with resultant inflammation, [20] however an additional role for *ARMS2* having a functional contribution cannot be ruled out [21].

With two main dichotomous pathways responsible for AMD, it has been hypothesized that a genetic approach to targeted therapy may yield a population most likely to benefit [22].

Gyroscope Therapeutics (a Novartis company) developed an ocular gene therapy for GA secondary to AMD which delivers an AAV2 vector-based *CFI* gene therapy to the site of disease using subretinal administration. PPY988 (formerly known as GT005) was targeted to provide a sustained expression of human FI in the retinal epithelium and photoreceptor cell layers of subjects with GA secondary to AMD, aiming to result in down-regulation of the complement AP and slow down macular degeneration [23].

Carriers of *CFI* type 1 RVs that lead to low FI levels were hypothesised to be a sub-group of GA most likely to respond to PPY988 as supplementing FI levels had the potential to restore complement system homeostasis, and slow down degeneration of the macula. The remaining *CFI* RVs associated with normal FI protein levels may confer reduced protein function as determined by *in vitro* assays (termed ‘type 2’) and patients with these variants may also be postulated to more likely benefit from FI supplementation. A proportion of the *CFI* RVs detected may also be functionally normal (benign). Assigning functional consequence is challenging for novel genotypes not previously described in the literature or clinical variant annotation databases with *in silico* analysis poorly representative of functional consequences. As such functional analysis of pure protein remains the gold standard to assess the nature of any dysfunction [24].

To increase chances of detecting a treatment response, one development strategy was to use the SCOPE natural history study (NCT03894020) to identify the ~3% of GA subjects positive for *CFI* type 1 RV and target them for enrichment into the PPY988 PhII clinical trial EXPLORE (NCT04437368). The remaining 97% were channelled to participate in the broader GA PhII trial HORIZON; (NCT04566445), where a treatment response to raising FI levels at the back of the retina via PPY988 gene therapy was also hypothesised, based on the widespread level of complement system dysregulation indicated by genetic studies [5, 25–28], with the complement proteins FI and FH being highlighted as particularly important. Lay et al demonstrated in vitro that supplementation of FI reversed the effects these AMD -risk complotypes had on AP activation [29]. Furthermore, the observation of complement proteins in drusen from AMD patients [30–32], and the growing number of clinical studies showing progression of atrophy is slowed by complement system inhibitors [33–35] also provide rationale for modulating the complement system in a broad GA population. However, following interim analysis of this large phase II trial (NCT04566445), the PPY988 therapy reached futility criteria [Press release: Novartis AG; September 11th 2023: https://www.novartis.com/news/gt005-ppy988-development-program-geographic-atrophy].

Prior to this, to support enrolment for the EXPLORE trial enriched for type 1 *CFI* RVs, extensive genotyping screening efforts were required to identify GA carriers of type 1 *CFI* RV. To support evaluation of potential locations for new trial sites, a genetic feasibility study was conducted in Finland to assess the *CFI* RV prevalence in the local dry AMD population.

This was driven in part by the knowledge that in Finland, strong bottlenecking events occurred around 200 years ago followed by rapid population expansion which have the potential to distort allele frequencies in unexpected ways [36]. Population bottlenecking resulted in numerous strongly deleterious alleles that occur more frequently in Finland compared to other European populations. Biobanks like the Finnish Biobank Cooperative (FINBB) and the public–private partnership research project FinnGen coupled with structured, digital health record data and extensive genotyping have been instrumental in uncovering how genetic allele frequencies are skewed in this isolated population and how this relates to health and disease [37].

To assess *CFI* RVs, DNA from a cohort of individuals with dry AMD accessed via the FINBB biobank were sequenced for *CFI* coding variants, and the resulting genotypes were assessed for type 1 or type 2 status according to previous annotations from the literature [9, 38]. Type 1 *CFI* RV genotypes were identified in Finnish dry AMD at a 5–10-fold lower level than found across European advanced AMD (0.212% versus 1.008% to 2.59%) [15], suggesting that genetic restriction in Finland has had an impact on the prevalence of *CFI* variants. This study also identified novel *CFI* RV genotypes enriched in FINBB dry AMD, which are now the subject of extensive in vitro functional characterization in this study to better understand their contribution to disease. This information sheds light on novel molecular mechanisms behind genetic dysfunction in *CFI*. Characterisation of novel genotypes adds to the growing list of dysfunctional *CFI* RVs, information which ultimately may help in the future clinical development strategies for complement inhibitors in AMD, and clinical diagnosis of other diseases associated with *CFI* RV mutations.

## Results

### Genetic analysis of CFI RVs identified in Finnish dry AMD

Targeted sequencing of *CFI* in 943 dry AMD subjects from FINBB identified 1.9% (n = 18/943) positive for a *CFI* RV that changed the coding sequence with a MAF (minor allele frequency) ≤ 1% in the Finnish European GnomAD database (v2.1.1, Table 1). A total of 7 different genotypes were identified (7/18; 38.8%), mirroring the same level of heterogeneity observed in other European AAMD datasets [14].

**Table 1:** Genetic variants changing the coding sequencing of *CFI* identified in a dry AMD cohort from FINBB. Genomic DNA and protein coordinates are provided alongside genotype reference SNP cluster ID (rsID). Number of *CFI* FINBB dry AMD variant genotypes and alleles are provided alongside control minor allele frequencies (MAF) for Finnish and non-Finnish Europeans, that were sourced from GnomAD V2.1.1. Odds ratio (OR) with *P* < 0.05 were considered statistically significant. *CFI* RV functional status according to the findings of this study and previous literature [9, 38]. n; number, CI; confidence interval, ND; not determined.

				**FINBB dry AMD**											
**Genomic coordinates**	**Variant rsID**	**Function**	**Protein change**	**Genotypes**			**Alleles**		**MAF**	**Finnish control MAF (%)**	**Non-Finnish European control MAF (%)**	**OR**	**95% CI**	**P value**	***CFI* RV functional status**
				**AA**	**Aa**	**aa**	**A**	**a**							
4-110662162-C-T	rs746522519	nonsynonymous SNV	G547R	942	1	0	1885	1	0.05302%	0.02786%	0.00000%	**1.9**	0.16 to 13.34	0.4396	Type 2
4-110667387-G-A	rs121964913	stopgain	R474X	942	1	0	1885	1	0.05302%	0.00000%	0.00616%	**34.44**	1.40 to 845.79	0.0302	Type 1
4-110 667 590-C-T	rs74817407	nonsynonymous SNV	R406H	899	43	1	1841	45	2.38600%	2.43500%	0.13330%	**0.97**	0.71 to 1.32	>0.9999	Normal
4-110 670 680-A-G	rs769419740	nonsynonymous SNV	I340T	941	2	0	1884	2	0.10604%	0.03696%	0.00792%	**2.87**	0.61 to 11.50	0.1884	Type 2
4-110 670 717-C-T	rs144164794	nonsynonymous SNV	G328R	941	2	0	1884	2	0.10604%	0.00000%	0.01320%	**57.43**	2.75 to 1196.79	0.0089	Type 1
4-110 681 527-C-T	rs112534524	nonsynonymous SNV	G261D	941	2	0	1884	2	0.10604%	0.01592%	0.19200%	**6.66**	1.26 to 28.61	0.0605	Normal
4-110 685 820-C-T	rs141853578	nonsynonymous SNV	G119R	942	1	0	1885	1	0.05302%	0.00798%	0.08519%	**6.64**	0.60 to 73.30	0.1221	Type 1
4-110 687 719-T-C	rs201419000	nonsynonymous SNV	T107A	934	9	0	1877	9	0.47720%	0.40200%	0.01940%	**1.18**	0.59 to 2.35	0.6215	Normal
Collective categorisation			Type 1	939	4	0	1882	4	**0.212089%**	0.00278%	ND	**72.62**	16.92 to 382.1	<0.0001	
			Type 2	940	3	0	1883	3	**0.159067%**	0.03207%	ND	**4.96**	1.522 to 15.74	0.0307	
			Normal	888	54	1	1830	56	**2.969247%**	0.95046%	ND	**3.18**	2.410 to 4.191	<0.0001	

Variant frequencies observed in FINBB dry AMD were compared to that found in Finnish individuals from the GnomAD database (n = 12 562, v2.1.1), and tested for association. GnomAD is a publicly available resource providing precise genetic variant allele frequency data from populations from different ethnicities and has been used as controls in genetic association studies when paired controls are unavailable [39]. In the FINBB dry AMD cohort, two novel *CFI* RV were identified encoding G547R and G328R, with G328R achieving significance (*P* = 0.0089, OR = 57.43, 95% confidence interval (CI) 2.75 to 1196.79).

**Figure 1:**
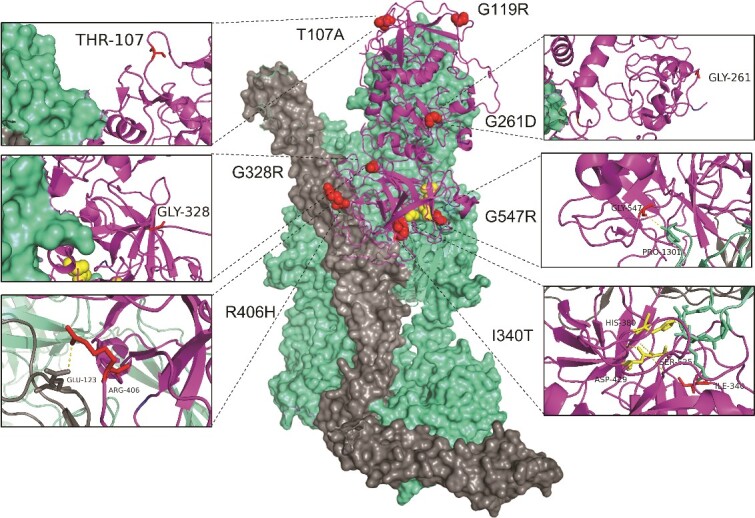
Structural modelling of rare genetic variants in FI described in the FINBB cohort on an x-ray–derived co-crystal structure of factor H CCP1-4_19–20/C3b/factor I displayed using PyMOL (V2.0.6, Schrödinger, LLC); FI (blue) bound to C3b (pale cyan) and FH CCPs 1-4_19-20 (dark grey). Red spheres highlight the rare genetic variants in FI and yellow spheres highlight the catalytic triad. Inserts demonstrate interactions of variant amino acids in FI with FH/C3b or the absence thereof. T107A, G261D and G328R are surface exposed and do not interact with either cofactor or substrate. The I340T variant destabilised the oxyanion hole at a catalytic triad interaction site. G547 would be predicted to interact with the scissile loop 1300–1306 of C3b at Pro130. The R406H variant resides in an unstructured loop in free FI however when complexed, interacts with E123 in CCP 2 of FH.

Compared to allele frequencies observed in non-Finnish Europeans (NFEs) (n = 64 603, v2.1.1), this study identified variants T107A, R406H and G261D at a greater prevalence rate in Finnish dry AMD and Finnish control data. Both R406H [9, 22, 38, 40] and G261D [9, 38, 40–43] have been extensively characterised with no loss of function demonstrated. Other previously characterised variants identified in FINBB dry AMD include the type 1 *CFI* RV G119R [8, 9, 11–13, 41, 43–45] and R474X [13, 38, 46, 47], and type 2 *CFI* RV I340T [22, 40]. Given the lack of functional data, G547R, G328R and T107A were taken forward for functional characterisation (Fig. 1).

### Expression and secretion of Finnish FI variants vs WT

**Figure 2:**
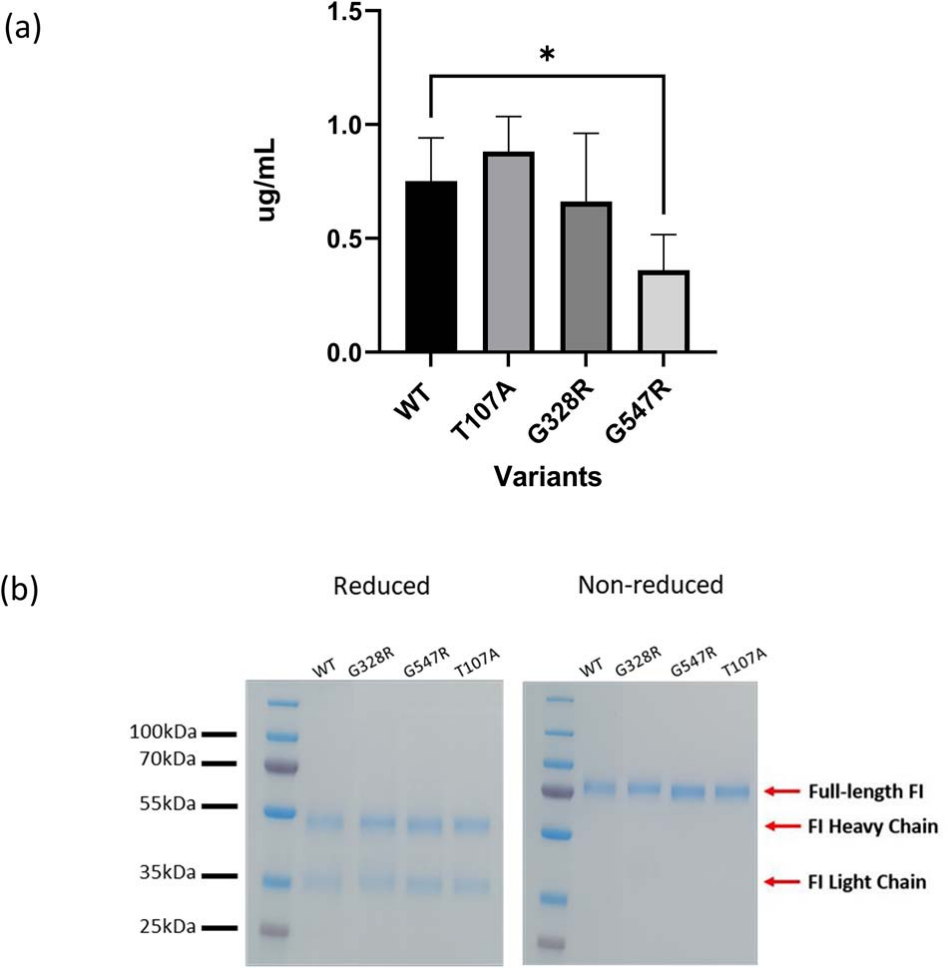
Recombinant expression of *CFI* rare genetic variants. (a) HEK293T cells were transfected with WT and mutant Furin-IRES-*CFI* expression vector and incubated for 72 h (n = 6/variant). HEK293T cells were transfected plasmids containing cDNA for each FI variant and incubated for 72 h in the absence of hygromycin. ELISA was used to determine the concentration [24]. The mean and standard deviation are displayed. Dunnett’s multiple comparisons test was used along with the standard one-way ANOVA test to compare the expression levels of the variants to the WT. Asterisks denote differences that are statistically significant (*P* < 0.05). (b) SDS-PAGE of purified recombinant FI under reducing and non-reducing conditions demonstrating fully processed FI with no evidence of unprocessed pro-I.

The 3 *CFI* RVs that were identified in the FINBB dry AMD cohort which lacked functional data (T107A, G328R, G547R) were selected for functional characterisation. In the absence of carrier serum to test circulating FI levels, in vitro assessment of mutant recombinant FI secretion was performed. All 3 variants were secreted albeit the G547R variant was secreted at slightly lower levels compared to wild type FI (Fig. 2a). Pro-I has previously been demonstrated to contaminate recombinant FI and being functionally inactive potentially confounds analysis [40]. All recombinant variants generated one band at 88 kDa in non-reducing conditions and two bands at 50 kDa and at 35 kDa in reducing conditions with no detectable Pro-I present, in keeping with complete post translational cleavage of the RRKR linker (Fig. 2b).

### Functional assessment of FI variants by fluid phase Co-factor assays of C3b cleavage

**Figure 3:**
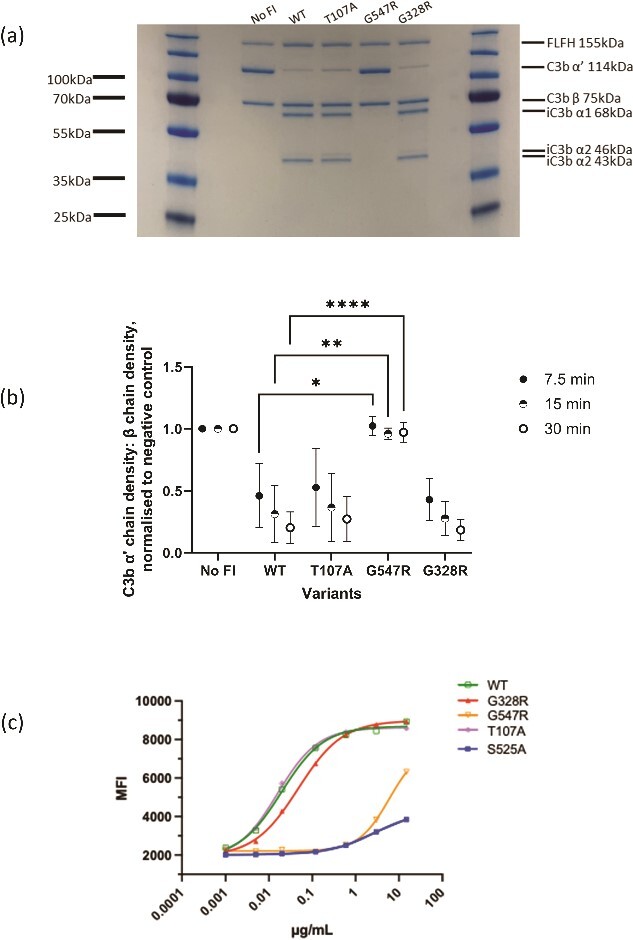
Characterisation of alternative pathway C3b regulatory activity of *CFI* variants. (a) Alternative pathway fluid phase cofactor assays for *CFI* variants. Separation of C3b products by SDS-PAGE followed by Coomassie staining was used to assess activity by the loss of the α′ band and generation of the iC3b α1 band (68 kDa) and α2 (46,43 kDa) bands (b) Alternative pathway kinetic fluid phase analysis of C3b cofactor activity for *CFI* variants. The density of C3b α′ chain remaining following 7.5, 15 and 30 min at 37°C was measured. The density of the α′ chain band was normalised to the density of the β chain band (loading control) before the resultant figure was normalised to a negative control containing no FI, giving a proportion of α′ chain remaining compared to the zero FI control. Fluid phase assays were repeated 3 times. Using a 2-way ANOVA multiple comparison test, the normalised density for each variant was provided as the mean ± SD and compared to the mean of the WT. (c) Solid phase cofactor assay. Each *CFI* RV was titrated in a 1:4 serial dilution and incubated with C3b-coated beads with excess FH for 1 h to allow cleavage of C3b. Four parameter logistic regression curves are shown by lines (WT: Green, T107A: Purple, G328R: Red, S525A: Blue). Each point shows the median fluorescence intensity (MFI) of a minimum of 1000 beads. The assay shown is representative of 3 independent repeats. FLFH: Full length FH.

To assess the AP regulatory ability of the FI variants, fluid phase AP co-factor assays were performed measuring proteolytic cleavage of the C3b α′ chain. There was a significant reduction in the regulatory activity of the G547R variant with no observable reduction in α′ chain or generation of breakdown products. No significant difference in regulatory activity was demonstrated for either T107A or G328R (Fig. 3a and b).

### Solid phase dynamic alternative pathway C3b functional assays of FI variants

To assess solid phase AP activity, a bead based functional assay (BBFA) of C3b breakdown was used [24]. IC50s for C3b cleavage to iC3b on beads revealed function similar to or moderately reduced compared to the WT protein (IC50: 0.01964 μg/ml) for the following variants: T107A (IC50: 0.01591 μg/ml) G328R (IC50: 0.04752 μg/ml). Meanwhile, variant G547R (IC50: 6.026 μg/ml) and the designed functionally inactive S525A mutant (IC50: 2.680 μg/ml), were shown to have substantial and significantly reduced proteolytic function for C3b in excess factor H, which is clearly demonstrated (Fig. 3c). Moreover, these results were consistent with the findings of the fluid phase co-factor assays for C3b wherein G547R, but not T107A or G238R, was functionally abrogated.

### Functional assessment of FI variants by fluid phase Co-factor assays of C4b cleavage

**Figure 4:**
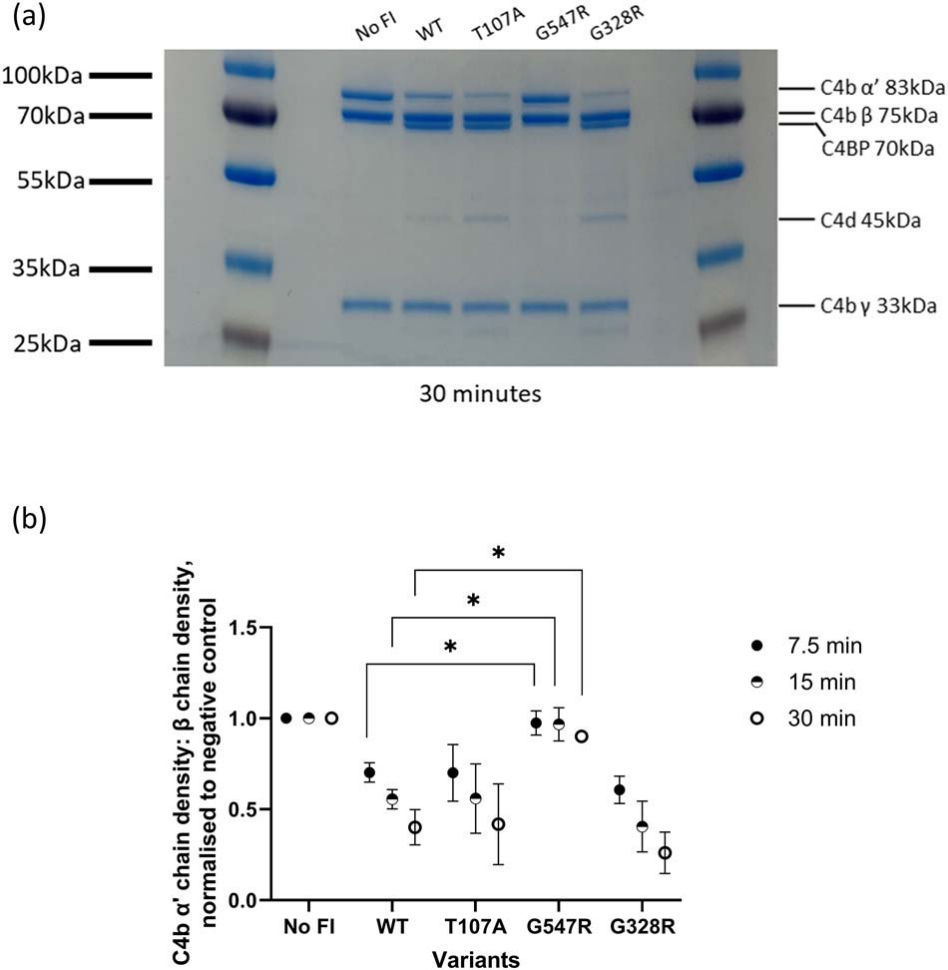
Characterisation of classical pathway regulatory activity of *CFI* variants. (a) Classical pathway fluid phase cofactor assays for FI variants. Separation of C4b products by SDS-PAGE followed by Coomassie staining was used to assess activity by the loss of the C4b α′ (83 kDa) band and generation of the C4d (45 kDa) bands. (b) Classical pathway kinetic fluid phase analysis of C4b cofactor activity for *CFI* variants. The density of C4b α′ chain remaining after incubation over a duration of 7.5, 15 and 30 mins at 37°C was measured. The density of the α′ chain band was normalised to the density of the β chain band (loading control), before the resultant figure was normalised to a negative control containing no FI, giving a proportion of α′ chain remaining compared to the zero FI control. Using a 2-way ANOVA multiple comparison test (n = 3), the normalised density for each variant was provided as the mean ± SD and compared to the mean of the WT.

Classical pathway (CP) regulatory activity was assessed using fluid phase CP co-factor assays detecting cleavage of C4b α′ chain. The results corresponded with the AP activity with a significant reduction in activity of G547R while no significant reduction in regulatory activity was demonstrated for either T107A or G328R (Fig. 4a and b).

### Minigene analysis reveals FI variant G328R alters splicing

**Figure 5:**
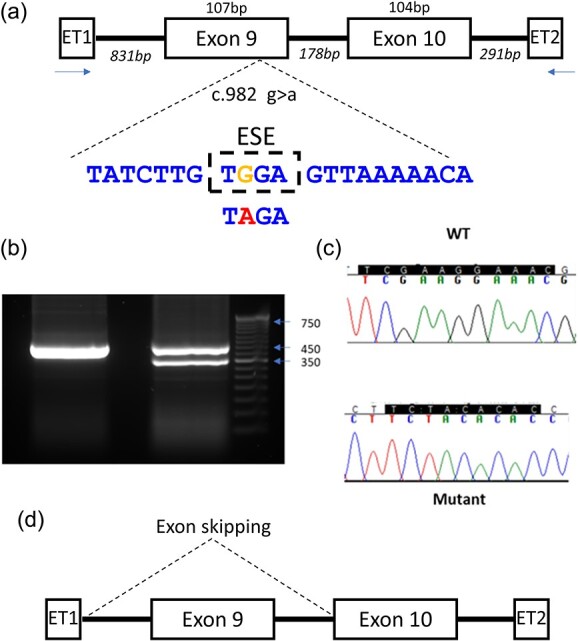
Modelling c.982 g>a, (G328R) using an exon trap vector. (a) The pET (MoBiTec) exon traps cloning vectors containing wild-type or mutated genomic DNA from intron 8 (IVS8-831) to intron 10 (IVS10 + 291). The putative exon splice enhance (ESE) and the c.982 g>a variant arehighlighted. (b) The wild type and mutant vectors were transfected into HEK293T cells and the products were analyzed by RT-PCR and run on a 2% agarose gel revealing a single band in the wild type and 2 bands in the c.982 g>a mutant. (c) Sequencing results of RT-PCR products. The RT-PCR products were subcloned into pCR2.1-TOPO and sequenced. The c.982 g>a variant was seen to produce a normal sequence and a second product (d) lacking exon 9 resulting in an out of frame deletion.

The finding that the FI variant G328R showed only a minimal reduction in function and normal expression from the cDNA plasmid in vitro despite conferring a significant level of disease predisposition (OR 57.43), led us to undertake further bioinformatic analysis to identify other underlying pathogenic mechanisms that may be driving a stronger phenotyping effect. In silico modelling using Alamut identified a potential alteration in gene splicing suggesting the genotype altered a splice enhancer sequence. In the absence of RNA from the carrier to test this hypothesis directly, a mini-gene spanning the region of interest using a pET-*CFI* exon trap vector was created. The wild type mini-gene demonstrated normal splicing of *CFI* however the G328R variant resulted in incomplete aberrant splicing with two different sized products. In addition to the normal splicing of exons 9 and 10, a second product lacking exon 9 was produced. Exon skipping resulting in an out-of-frame deletion of exon 9 is predicted to result in FI haploinsufficiency, due to resulting transcript degradation via nonsense mediated decay (Fig. 5).

### Combined odds ratios for CFI RV identified in Finnish AMD categorized as type 1, type 2 or normal function

New and existing functional evidence was summarized to provide a final categorization for each of the *CFI* RV identified in Finnish GA in AMD (Table 1). Collectively the type 1 *CFI* RVs associated with FI low levels or haploinsufficiency were significantly enriched with increased odds for dry AMD of 72.6 (95% CI 16.92 to 382.1). The *CFI* RV assigned as type 2, describing genotypes associated with FI dysfunction but normal FI levels, collectively conferred a significant OR for dry AMD of 4.97 (95% CI 1.522 to 15.74), *CFI* RV assigned as non-impaired with no evidence of dysfunction collectively conferred an increased odds of 3.19 (95% CI 2.410 to 4.191). This magnitude of enrichment observed in Finnish dry AMD for each category of *CFI* RV was much greater than that observed in a previous European advanced AMD study that undertook extensive targeted sequencing of *CFI* [9] Individual rare variant frequences of *CFI* RV genotypes from the European advanced AMD study that were identified in FINBB, were combined into the respective type 1, 2 and Normal genotype groups to derive collective ORs, however none proved statistically significant when compared to frequencies observed in matched European controls (Supplementary Table 1).

## Discussion

FI is the master complement regulatory enzyme downregulating CP, Lectin pathway and AP of complement [23]. Complete FI deficiency has been associated with a consumptive C3 deficiency and recurrent infections with encapsulated microorganisms [23] or aseptic cerebral inflammation [48, 49] while heterozygous mutations in *CFI* have been liked to immunopathology in the form of atypical haemolytic uraemic syndrome [50, 51] and AMD [7, 8].

The unique genetic make-up of the Finnish population resulting in the increased frequency of certain variants has made it attractive for undertaking genotype/phenotype studies [52]. The effects of this genetic restriction can be detected in the variation at *CFI* when comparing background allele frequencies in Finnish and Non-Finnish European control populations.

Altered frequencies of genetic factors that are adversely associated with increased disease risk could theoretically be expected to alter the overall incidence of disease at a population level depending on strength of selection pressure and demographic history of the population [53–55]. Recent investigations into the prevalence of GA in a study examining Nordic countries that included Finland (alongside Denmark, Iceland, Norway, and Sweden) were estimated to be 0.4% (95% confidence intervals [CI]: 0.2%–0.8%), 1.5% (95% CI: 0.7%–2.6%), and 7.6% (95% CI: 4.6%–11.3%) for individuals aged 60–69, 70–79, and 80+ years, respectively [56]. The prevalence of another form of late AMD, neovascular age-related macular degeneration, in the Finnish population was 3% in those aged between 75–96 years [57]. The prevalence of both forms of late AMD reported in Finland do not appear markedly different to that collectively measured across many European cohorts, where late AMD was estimated to be 0.41% (95% CI: 0.27–0.60), 1.71% (95% CI: 1.17–2.44), and 4.56% (95% CI: 2.96–6.73), for those aged 60–69, 70–79, and 80+ years, respectively [1]. Any apparent differences reported here in prevalence of *CFI* RVs in the Finnish population are unlikely to have altered the overall epidemiology of AMD, which is to be expected given AMD is a common disease with a complex aetiology influenced by both genetics and many lifestyle-related and environmental factors that develops mostly in late adulthood [58]. One limitation of this study was the use of health registry ICD10 codes by the biobank which were then utilised to identify individuals with dry AMD for *CFI* sequencing. The ICD10 codes were assigned by the subject’s respective health care provider and logged in the healthcare management system before information was added to the biobank database. A formal assessment of disease status and grade was not undertaken by a retinal specialist to confirm this diagnosis specifically for this study, therefore we cannot rule out miss-diagnosis and potentially inclusion of individuals that did not have dry AMD.

There is a greater prevalence for the *CFI* RVs R406H and T107A (19-fold and 20-fold, respectively) in Finnish controls. To contrast, G261D frequency is 5-fold less frequent in Finnish controls compared to Non-Finnish European counterparts, however is enriched in Finnish dry AMD (OR = 6.66, 1.26 to 28.61, 95% CI *P* = 0.06) despite being documented as having no clear evidence for protein dysfunction or haploinsufficiency [11, 38, 40].

Previous analysis of the Finnish AMD cohort had focused on *CFI* RVs known to cause low levels of FI in the circulation (type 1 variants) in multiple studies. Enrichment of type 1 *CFI* RVs in AMD in Finland was said to be driven by only 2 rare variants, G119R and R474X [14]. To provide granularity on the Finnish population we undertook functional analysis of all variants where the classification was uncertain and because there were no serum or plasma samples available in the biobank from carriers of these *CFI* RV genotypes to measure FI levels directly.

The c.982 g>a variant encoded a G328R amino acid change and recombinant generation of this mutant demonstrated FI levels in culture supernatant equivalent to wild-type protein. Functional analysis of this variant also revealed normal regulatory function in both CP and AP assays. Although this variant did not reside in the canonical consensus donor and acceptor splice site sequences, we undertook bioinformatic analysis to look for potential perturbations of intra-exonic sequences which may assist splicing. This suggested that the c.982 g>a variant altered an exonic splicing enhancer site. Exonic splicing enhancers (ESEs) activate nearby splice sites and promote inclusion of the exon in which they are present, however they are very difficult to predict [59, 60]. In the absence of patient RNA, we generated an exon splice vector containing the genetic region surrounding the variant to definitively establish if the loss of this putative ESE altered splicing. This confirmed the variant did result in some skipping of exon 9 and an out of frame deletion which would lead to nonsense mediated decay. Subsequent to our analysis an individual with the variant has been reported to have low levels of FI [61]. This highlights a problem with the use of in vitro assays of protein production to detect type 1 mutations as cDNA will obfuscate the detection of altered splice consensus sequences. This may result in the misclassification of rare genetic variants even following detailed functional analysis. As such we can classify the c.982 g>a (G328R) as a type 1 variant.

This allowed us to reanalyse the effect of type 1 variants in AMD in Finland revealing an OR of 72.6 (95% CI 16.92 to 382.1).

Recently a rare variant located in the non-coding part *CFI* was identified as significantly enriched in Finnish AMD [37]. The variant rs139779213 is located in the 3′ untranslated region (UTR) of *CFI* and increased risk of AMD in Finnish population (OR 1.1, *P* = 1.8 × 10–7). The variant was found in 2% Finnish AMD (n = 3763), but not detected in 404 535 Europeans in the UK Biobank with or without AMD (n = 3298 and 404 535, respectively). The functional nature of this non-coding variant is not fully elucidated however it has been linked to reduced *CFI* transcription, a plausible mechanism that may explain correlation with disease [62, 63]. We were unable to assess the frequency of rs139779213 in this study due to its location being outside the coding region captured by targeted sequencing. Our finding that another Finnish specific *CFI* variant G328R likely affects splicing is another example of non-canonical mechanisms for causing FI dysfunction in AMD. Verifying the predicted functional impact in carriers of these genotypes by testing systemic protein levels would help confirm these mechanisms also lead to haploinsufficiency, and the variants can be confirmed as type 1.

A single type 2 variant, I340T, had been reported in the Finnish AMD cohort. Previous analysis by ourselves and others revealed that the I340T variant destabilised the oxyanion hole at a catalytic triad interaction site (Fig. 1) [22, 40]. This resulted in loss of AP and CP regulatory activity of FI. The G547R variant resides in a loop in the serine protease domain of FI and is highly conserved. G547 would be predicted to interact with the scissile loop 1300–1306 of C3b at Pro1301 (Fig. 1). Consistent with this, G547R has almost entirely abrogated ability to cleave C3b or C4b and can be classified as a type 2 variant. Analysis of the two type 2 *CFI* variants in the AMD Finnish population revealed a combined OR of 4.97 (95% CI 1.522 to 15.74).

Functional analysis of T107A failed to detect any defect in either secretion or AP or CP regulatory activity. Serum levels are not reduced. This lack of functional effect may be predicted given the surface associated amino acid does not interact with C3b or FH on the crystal structure of the AP regulatory trimolecular structure. Likewise the G261D variant is surface exposed and not predicted to interact with either cofactor or substrate (Fig. 1). Multiple studies both in aHUS and AMD have demonstrated normal serum levels of the G261D protein and no statistically significant effect on function [9, 11–13, 38, 40–43, 64, 65].

The R406H variant resides in an unstructured loop in free FI however when complexed, interacts with E123 in CCP 2 of FH. Despite this, only non-significant effects on function have been demonstrated and recombinant protein generation and serum FI levels are consistently normal [9, 11, 38, 40, 43, 45]. It was not enriched in AMD in other non-Finnish cohorts [9].

Collectively these unperturbed variants (T107A, G261D and R406H) have an OR of 3.19 (95% confidence interval 2.410 to 4.191) consistent with their limited functional effect. Almost all the effect of the variants with no detectable functional consequence is driven by G261D. The variant has been described in other AMD non Finnish AMD cohorts with no enrichment seen in disease [9]. We cannot rule out that this variant is in linkage disequilibrium with a different locus which is responsible for the increased odds ratio in this Finnish population.

The small number of *CFI* RVs identified in this Finnish population allowed functional studies of the variants to provide a complete overview of effects of *CFI* in disease in Finland. Type 1 variants carry the highest odds ratio for AMD 72.6, with type 2 variants having an odds ratio of 4.97 with variants where no functional effect could be established having the lowest OR for disease. A review of the *CFI* genotypes identified in Finnish dry AMD indicated they were not significantly enriched in a previous study of European AAMD cases and matched European controls [9]. This could potentially indicate some of the rare variant genotypes identified could be restricted to Finnish population, however much greater powered studies are required to fully investigate this.

Our study also demonstrated the effects coding variants can have in non-canonical splicing elements which generation of recombinant protein with cDNA may fail to detect.

## Materials and Methods

### Methods

#### Patient cohort

Using health registry ICD10 code data, 943 individuals were identified with dry AMD (excluding wet AMD, choroid neovascularization, glaucoma or diabetic retinopathy), who were currently alive and consented for recontacting, and DNA samples were available across the biobanks within the Finnish Biobank network as described [14].

#### Genetic analysis

Sequencing of the *CFI* coding region was performed as previously described by the Finnish Institute of Molecular Medicine laboratory [14]. Variant in silico splicing predictions were undertaken using Alamut (V2.12).

#### Genetic statistical analysis

Variant positions were provided to genome build hg19/GRCh37 coordinates, and FI protein accession number NP_000195.2. Genotypes were annotated with minor allele frequencies (MAFs) from Finnish Europeans and non-Finnish Europeans (NFE) in GnomAD (V2.1.1). Data were accessed through the gnomAD browser (http://gnomad.broadinstitute.org) and the term ‘observed Afs’ was used as the value representing the count ratio of the actually detected minor alleles to reliably sequenced alleles. For each genotype, the MAF was calculated as the number of *CFI* RV alleles divided by total alleles. The association of *CFI* RV with dry AMD was calculated using Fisher’s exact test. OR confidence intervals were derived using Woolf logit interval calculation. For each functional categorisation (type 1, type 2 or normal) the number of alleles for genotypes assigned to that category were summed and divided by the total number of alleles for genotypes assigned to that category to generate the combined MAF, and the respective MAFs for dry AMD were compared to that from Finnish Europeans (GnomAD) to test for significant association of *CFI* RV with dry AMD. Statistics was conducted using GraphPad PRISM (v10.1.2). Instances of zero values for alleles in gnomAD were converted to 0.5 using Haldane-Anscombe to compute the OR [66, 67].

#### Recombinant protein production and purification

Recombinant FI was produced using the methodology previously described [22]. In brief, site-directed mutagenesis (SDM) was carried out using the Agilent QuickChange XL Site-Directed Mutagenesis kit (Agilent, 200517) on a wild type *CFI* IRES vector containing *CFI* and furin to generate *CFI* variants (T107A, G328R, G547R). The primers were: for **T107A** forward (f) CCAGGGACAAAGTTTTTAAATAACGGAACATGCGCAGCCGAAGGAAAGTTTAGTGTTTCC,reverse (r) GGAA ACACTAAACTTTCCTTCGGCTGCGCATGTTCCGTTATTTAAAAACTTTGTCCCCTGG, for **G328R** (f) GGATAAA ATCATTATTACCTAAACTACTTGTAGAGTTAAAAACAGAATGCACATTCGAAGG, (r) CCTTCGAAGTGCATTCT GTTTTTAACTCTACAAGATAGTTTAGGTAATAATGATTTTATCC, and for **G547R** (f) GGGGTGTTGTGAGTTG GAGGGAAAACTGTGGAAAACCAGAGTTCCCAGGTG, (r) CACCTGGGAACTCTGGTTTTCCACAGTTTTCCCTC CAACTCACAACACCCC. Plasmid DNA containing the mutations and the WT were transfected to HEK293T cells with JetPEI™ reagent (Polyplus, Illkirch, France). The ÄKTA Start (GE) protein purification system was utilised for FI purification. The supernatant was loaded onto a 1 ml HiTrap NHS-activated HP column (Cytiva, 17071601), containing the OX-21 monoclonal antibody produced in house. The column was equilibrated with PBS with 0.01% sodium azide to remove any unbound protein. Using 0.1 M Glycine, pH 2.7, the bound FI was then eluted into 1 ml fractions containing 200 μl 1 M Tris (pH 9.0) for neutralisation. The peak fractions of each protein were collected after purification, and they were buffer exchanged into PBS using PD-10 columns (Cytiva, 17085101). To identify the protein’s size, the purified FI was run on 10%–20% Sodium dodecyl sulphate-polyacrylamide gel electrophoresis (SDS-PAGE) gels and visualised using InstantBlue Coomassie Protein 51 Stain (Sigma). FI protein expression and concentration was also determined using an ELISA as per Hallam *et al* [24]. In brief a polyclonal antibody sheep anti-human FI (LS-C147802, LSBio) was coated at 2 μg/ml. A monoclonal OX-21 antibody was used for detection, followed by an HRP-conjugated donkey anti-mouse secondary ab (715-035-150JIR, Stratech).

#### Fluid phase Co-factor assays

C3b fluid phase assays were carried out using the following amounts of components: full length factor H (Comptech), 250 ng; C3b (Comptech), 1000 ng and factor I (FI), 18.8 ng. For C4b fluid phase assays, the following amounts were used: C4b binding protein (Comptech), 250 ng; C4b (Comptech), 1500 ng and FI, 18.8 ng.

All dilutions were made in PBS and as a negative control, full-length factor H and C3b, or C4BP and C4b were used respectively but no *CFI*. All reactions were kept on ice and were then placed in a 37°C water bath. Samples were removed at 7.5, 15 and 30 min. and the reactions were stopped using 5 × Lane Marker Reducing buffer (Thermo Scientific), and heated at 95°C for 5 min. The products were then visualised using Coomassie staining on a 10%–20% SDS-PAGE gel.

#### Assessment of FI variants by bead based functional assay of C3b breakdown

Human C3b protein (A114, CompTech, Tyler, TX, USA) was biotinylated using EZ-Link Melamide-PEG2-Biotin (Thermo Scientific, A39261) following the manufacture’s protocol. Beads were prepared coating 4.5 × 10^7^ beads/ml of M-270 Streptavidin Dynabeads (ThermoScientific, 65305) with biotinylated human C3b (2.5–5 μg/ml) for 1 h at RT with continuous mixing. Next, C3b coated beads were plated in a 96 well plate at a final concentration of 1 × 10^7^ beads/ml and washed twice using an automated plate washer twice with PBS-0.05% Tween 20. The beads were incubated with FI (WT or variant) titrated with eight 1:4 dilutions at 15 μg/ml final concentration, the dilution solution was spiked with an excess (16 or 20 μg/ml) of complement factor H (A137, CompTech, Tyler, TX, USA) Samples were incubated at 37°C with shaking at 750 RPM for 1 h. Beads were washed as previously described and incubated with a high salt buffer (1% BSA, 1 M NaCl in PBS) at room temperature with shaking for 20 mins to remove bound FH. The beads were washed and blocked in 5% BSA in Cell stain buffer (Bio-legend, 420201) before staining with a murine monoclonal anti-iC3b antibody (Quidel, A209; used at 1:1000) with shaking at RT for 30 mins. The plate was washed as previously described and incubated with chicken anti-mouse A488 (ThermoScientific, A-21200, 1:100). The beads were washed and re-suspended in 120 μl 0.5% BSA in PBS for flow cytometric analysis (BD Lyric). Immuno-stained beads were probed with a blue laser at 488 nM with 527/32 filters to measure A488 fluorescence intensity, gating was used to isolate single bead populations avoiding inclusion of bead aggregates in the analysis. Median fluorescence intensities (MFI) were obtained and fitted using 4PL (4 parameter logistic regression model) curves using GraphPad Prism V9 to generate IC50s.

#### Mini-gene experiment

To generate a *CFI* minigene for analysis of the effect of G328R in splicing, a forward primer was designed before exon 9 (GCGGCCGCGGGCACAAGACTGAGACTCC) of the *CFI* gene as well as a reverse primer just after exon 10 (CGCCGGCGTGTGGGGCAAGATGAGATTGG) introducing NotI restriction sites.

The control genomic DNA was amplified by PCR, followed by gel electrophoresis in denaturing conditions as previously described by Caprioli *et al*. [68]. The PCR product was subcloned into pCR™-Blunt II-TOPO® (Invitrogen, Carlsbad, CA). The mutant was produced by the Agilent QuickChange XL Site-Directed Mutagenesis kit (Agilent, 200517) using primers (F) C CTA AAC TAT CTT GT**A** GAG TTA AAA ACA GAA TGC ACA TTC G and (R) C GAA TGT GCA TTC TGT TTT TAA CTC TAC AAG ATA GTT TAG G. Mutant and wild-type DNA fragments were cloned into the exon trap cloning vector pET (MoBiTec, Goettingen, Germany) [68].

Transfections were performed into HEK293T cells and the RNA was extracted 72 h later. The mRNA was extracted from the 293T using the RNeasy mini kit (Qiagen Inc., Hilden, Germany) and by following the manufacturer instructions. Reverse transcription–PCR (RT-PCR) (Qiagen Inc., Hilden, Germany), was performed using a forward primer constructed in ET1 (5′—GATCGATCCGCTTCCTG CCCC- 3′) and a reverse primer in ET2 (5′—CTGCCGGGCCACCTCCAGTGCC—3′) (Fig. 5a). The amplicons were sequenced either directly or after cloning in E coli. After RT-PCR, the cDNA products were separated on 2% agarose gel and subcloned into pCR2.1-TOPO for sequencing.

### Statistics

Densitometry analysis of SDS-PAGE gels for fluid phase cofactor assays was carried out using Image studio V5.2 (Licor, UK). Data were analyzed using GraphPad Prism v9 (GraphPad Software, San Diego, CA; www.graphpad.com). T-tests were used to compare each variant to the WT in fluid phase co-factor assays. For the BBFA, IC50s were calculated using a 4-parameter fit logistic regression curve. Statistically significant results are indicated by (^*^), (^*^^*^) or ^*^^*^^*^) and defined as ^*^P < 0.05, ^*^^*^P < 0.01, ^*^^*^^*^P < 0.001.

## Supplementary Material

Supplementary_Table_1_ddae165

## Notes

*Conflict of interest statement*: D.K., Gyroscope Therapeutics (consultancy, equity, grant income), Alexion Pharmaceuticals (consultancy), Novartis (consultancy), Apellis (consultancy), Samsung (consultancy), Amgen (consultancy); C.L.H.: Q32 Bio (consultancy, employee, equity), Gyroscope Therapeutics (consultancy), Novartis (employee), Ra Pharmaceuticals (grant income), Biocryst (consultancy); T.M.H.: Gyroscope Therapeutics, Novartis (employee); S.J.S.: Gyroscope Therapeutics, Novartis (emoloyee); EG: Gyroscope Therapeutics, Novartis (employee); ad: Gyroscope Therapeutics, Novartis, Beacon Therapeutics (employee); A.V.J.: Gyroscope Therapeutics, Novartis (employee). A.L.: Gyroscope Therapeutics (consultancy, equity), Roche (consultancy), Apellis (consultancy), Novartis (consultancy) K.J.M.: Qualasept (consultancy), Freeline Therapeutics (consultancy), Catalyst Biosciences (consultancy, grant income), Idorsia Pharmaceuticals (grant income), Gemini Therapeutics (consultancy, grant income), Alexion Pharmaceuticals (grant income, consultancy). A.A; V.B; P.R.W declare no conflict of interest.
