# Supplementary material for: The role of complement factor I rare genetic variants in age related macular degeneration in Finland
Source: Hum Mol Genet. 2024 Nov 25;34(3):218–28. doi: 10.1093/hmg/ddae165 (PMC11792236; doi:10.1093/hmg/ddae165)
Supplement: Supplementary_Table_1_ddae165 [file supplementary_table_1_ddae165.docx]

**Supplementary Table 1.**

|  |  |  |  | **European AAMD (n=2266)** | | | **Matched European controls (n=1400)** | | |  |  |  |
| --- | --- | --- | --- | --- | --- | --- | --- | --- | --- | --- | --- | --- |
| **Genomic coordinates** | **Variant rsID** | **Final CFI RV functional status** | **Protein change** | **Alleles** | |  | **Alleles** | |  | **OR** | **95% CI** | **P value** |
|  |  |  |  | **A** | **a** | **MAF** | **A** | **a** | **MAF** |  |  |  |
| 4-110662162-C-T | rs746522519 | Type II | p.G547R | 4532 | 0 | 0.000% | 2800 | 0 | 0.000% | 0.618 | 0.012 to 31.149 | 0.809 |
| 4-110667387-G-A | rs121964913 | Type I | p.R474X | 4530 | 2 | 0.044% | 2800 | 0 | 0.000% | 3.091 | 0.148 to 64.404 | 0.466 |
| 4-110667590-C-T | rs74817407 | Normal | p.R406H | 4531 | 1 | 0.022% | 2794 | 6 | 0.214% | 0.103 | 0.012 to 0.854 | 0.035 |
| 4-110670680-A-G | rs769419740 | Type II | p.I340T | 4531 | 1 | 0.022% | 2800 | 0 | 0.000% | 1.854 | 0.075 to 45.530 | 0.705 |
| 4-110670717-C-T | rs144164794 | Type I | p.G328R | 4531 | 1 | 0.022% | 2800 | 0 | 0.000% | 1.854 | 0.075 to 45.530 | 0.705 |
| 4-110681527-C-T | rs112534524 | Normal | p.G261D | 4520 | 12 | 0.265% | 2792 | 8 | 0.286% | 0.927 | 0.378 to 2.269 | 0.867 |
| 4-110685820-C-T | rs141853578 | Type I | p.G119R | 4522 | 10 | 0.221% | 2798 | 2 | 0.071% | 3.094 | 0.677 to 14.130 | 0.145 |
| 4-110687719-T-C | rs201419000 | Normal | p.T107A | 4532 | 0 | 0.000% | 2800 | 0 | 0.000% | 0.618 | 0.012 to 31.149 | 0.809 |
|  |  |  | Type I | 4519 | 13 | 0.287% | 2798 | 2 | 0.071% | 4.013 | 0.904 to 17.796 | 0.067 |
|  |  |  | Type II | 4531 | 1 | 0.022% | 2800 | 0 | 0.000% | 1.854 | 0.075 to 45.530 | 0.705 |
|  |  |  | Normal | 4519 | 13 | 0.287% | 2786 | 14 | 0.500% | 0.573 | 0.268 to 1.219 | 0.148 |

**Minor allele frequency data of rare *CFI* genetic variants in a European AAMD cohort, previously reported by Kavanagh et. al (2015).** Only variant frequency data from *CFI* rare variants identified in FINBB dry AMD are provided individually and collectively for each final functional designation (Type I, Type II or Normal), to act as a comparison to prevalence in Finnish dry AMD. Genomic DNA and protein coordinates are provided alongside genotype Reference SNP cluster ID (rsID). Number of *CFI* variant alleles are provided alongside control minor allele frequencies (MAF) from AAMD cases and European controls (ref). Odds ratio (OR) with P<0.05 were considered statistically significant, CI; confidence interval.
